# Editorial: Lifestyle and environmental influences on Alzheimer's disease: exploring the roles of diet, exercise, cognitive reserve, sleep, and air quality

**DOI:** 10.3389/fnagi.2025.1657074

**Published:** 2025-07-29

**Authors:** Ruogu Cheng, Song Qiao, Hongquan Wang, Yan Liu, Zhengjun Wang, Guohao Wang, Pei Shang

**Affiliations:** ^1^Department of Neurology, Nanfang Hospital, Southern Medical University, Guangzhou, China; ^2^Department of Neurology, Zhejiang Hospital, Hangzhou, China; ^3^Aerospace Center Hospital, Peking University Aerospace School of Clinical Medicine, Beijing, China; ^4^School of Pharmacy, Lanzhou University, Lanzhou, China; ^5^Department of pharmacology, Addiction Science and Toxicology, University of Tennessee Health Science Center, Memphis, TN, United States; ^6^Synapse and Neural Circuit Research Section, National Institute of Neurological Disorders and Stroke, National Institutes of Health, Bethesda, MD, United States; ^7^Key Laboratory of Animal Models and Human Disease Mechanisms of the Chinese Academy of Sciences & Yunnan Province, Kunming Institute of Zoology, Kunming, Yunnan, China; ^8^Department of Neurology, Mayo Clinic, Rochester, MN, United States

**Keywords:** Alzheimer's disease, environmental condition, lifestyle, cognition, neurodegeneration

Alzheimer's disease (AD) is a neurodegenerative disorder characterized by a gradual onset and a protracted course, and it is the leading cause of dementia. The primary pathological features of AD include the accumulation of β-amyloid plaques and the hyperphosphorylation of Tau protein. In patients with AD, the accumulation of β-amyloid initially occurs in the preolfactory cortex. This deposition progressively extends to the entorhinal cortex and finally encompasses the associated cortical regions of the frontal, parietal, and temporal lobes. Further, the hyperphosphorylation of Tau protein significantly impairs the structural integrity of neurons. This leads to degeneration and subsequent loss of a large number of neurons, resulting in brain atrophy within the hippocampus and cortex, which gradually deteriorates as the disease progresses and the aging process. Currently, AD has become the most rapidly increasing neurodegenerative disorder in the world, imposing a huge burden on both families and society.

So far, the available treatment modalities for AD are still constrained. Previous research has demonstrated that conventional pharmacological agents, including cholinesterase inhibitors and NMDA receptor channel antagonists, can enhance the clinical manifestations of AD and mitigate its progression (Rijpma et al., 2014; Johnson and Kotermanski, 2006). Nevertheless, these therapeutic interventions cannot provide a cure for AD, exhibit minimal effect on the underlying pathological function of the disease, and thus present challenges in managing the long-term progression of AD (Zhang et al., 2025). Furthermore, although the FDA has recently approved monoclonal antibodies aimed at β-amyloid for the treatment of early symptomatic AD, the current experience with anti-β-amyloid disease-modifying therapies (DMTs) is still limited. Consequently, further research is essential to evaluate both the clinical efficacy and the economic implications of these therapies.

Mild cognitive impairment (MCI) associated with AD represents an intermediary phase between cognitive health and the onset of AD (Hoang et al.) This stage serves as a “window period” for early diagnosis and prevention of AD. Neurobiologically, it is marked by reduced blood flow and metabolic activity in the temporoparietal cortex, atrophy of the medial temporal lobes—especially in the nasal cortex—elevated Tau protein levels in cerebrospinal fluid (CSF), diminished phosphorylation and Aβ_42_ levels, as well as the deposition of Aβ_42_ in the brain. Clinical manifestations of MCI include symptoms of depression as well as the utilization of avoidance coping strategies (Anderson, 2019). Delaying the progression of MCI to AD will effectively reduce the incidence of AD and result in substantial savings in medication expenses. This has prompted an examination of the influence of risk factors, such as lifestyle and environmental elements, on the progression of AD, as well as efforts to mitigate the risk of AD through multifaceted intervention strategies. In addition, it has a notable advantage that cannot be ignored compared to other drug therapies, as it is non-toxic and does not cause any negative side effects.

Advanced age has been widely recognized as the most important risk factor for the development of AD. At the same time, the other 12 modifiable factors, including cardiovascular health and poor dietary patterns, have been gradually demonstrated to be associated with an increased risk of AD. The combined rate of these potential risks of AD due to these factors is 40% globally, which is a significant number, suggesting that intervention with these pre-adjusted risk factors is crucial for preventing AD (Scheltens et al., 2021).

Despite this, there are few empirical studies on the use of non-pharmacological interventions for the prevention and treatment of AD worldwide. In this Research Topic, we focused on describing the lifestyle and environmental influences on AD, especially exploring the roles of diet, exercise, cognitive reserve, sleep, and air quality. A total of 19 articles on this topic have been published, primarily summarizing the potential impact of the intervention of lifestyle and environment on the progression of AD. Recent research has illuminated the intricate interplay between lifestyle and environmental factors in modulating AD risk and progression. Diet, particularly adherence to the Mediterranean diet, significantly reduces cardiovascular-related mortality among cognitively impaired individuals, suggesting a potential protective role in AD pathways (Li L. et al.; Wang et al.) Exercise is repeatedly shown to bolster cognitive resilience, with a frequency of ≥3 times/week, offering optimal benefits. Notably, combining physical activity with natural neuroprotective compounds, such as platycodin D, has synergistic effects in reducing amyloid burden and inflammation in AD mouse models (Liu et al.).

Cognitive reserve (CR), shaped by education and possibly enhanced by brain clearance systems like the glymphatic pathway, emerges as another critical factor (Zhou et al.) Higher education independently correlates to reduced risk of cognitive impairment, and glymphatic activity appears to mediate CR's benefits on cognition. Tools like nomograms incorporating CR, age, and genetic predispositions also show promise for early identification of MCI (Zhong et al.).

Sleep duration interacts with metabolic health to influence cognitive outcomes, especially in overweight and obese older adults, where a sleep window of 5–6 hours may be neuroprotective (Qiu et al.). Similarly, environmental exposures such as secondhand smoke (SHS) and pollutants like acrolein have been implicated in accelerating cognitive decline, particularly when combined with other vulnerabilities like vitamin D deficiency (Li Y. et al.). Acrolein's role in promoting oxidative stress and amyloid-beta toxicity underscores the impact of air quality on AD pathology (Jallow et al.).

Moreover, rural living and neighborhood disadvantages have been associated with distinct neuroanatomical changes, pointing to broader socioeconomic and environmental contributors to AD risk (Zhuang et al.). Additional insights highlight that metabolic factors such as high BMI and elevated cardiac metabolic index (CMI) are biomarkers of accelerated aging and potential contributors to AD (Sun and Bao.).

Finally, entertainment-based cognitive activity, such as computer use, shows a modest but significant protective association, reinforcing the value of sustained mental engagement (Lu et al.). As dementia continues to impose a significant global burden, this growing body of evidence emphasizes the multifactorial nature of AD and supports comprehensive prevention strategies targeting modifiable lifestyle and environmental factors (Zhong et al.; Sun et al.; Wen et al.).

In summary, based on our topic manuscripts, we propose that clinicians should be aware that AD can be prevented, and the progression of this incurable disease may be delayed through the modification of assorted risk factors, although the causal relationship between these non-pharmacological interventions and the progression of AD still needs to be confirmed in large-scale studies and national reports.
